# Genetic analysis suggests extensive gene flow within and between catchments in a common and ecologically significant dryland river shrub species; *Duma florulenta* (Polygonaceae)

**DOI:** 10.1002/ece3.5310

**Published:** 2019-06-17

**Authors:** Bruce Murray, Michael Reid, Samantha Capon, Shu‐Biao Wu

**Affiliations:** ^1^ Geography and Planning, Faculty of Humanities Arts and Social Sciences University of New England Armidale New South Wales Australia; ^2^ Australian Rivers Institute Griffith University Nathan Queensland Australia; ^3^ School of Environmental and Rural Science University of New England Armidale New South Wales Australia

**Keywords:** dispersal, dryland rivers, *Duma florulenta*, gene flow, genetic structure, keystone species, microsatellites, riparian vegetation

## Abstract

**Aim:**

The conservation of plant species biodiversity has been identified as a crucial factor for the resilience of dryland ecosystems in the face of climate change and desertification. *Duma florulenta* (lignum) is a keystone species that facilitates biodiversity in the floodplains and wetlands of Australia's dryland river systems. This paper explores spatial genetic structure of lignum and investigates factors influencing dispersal and gene flow within and among river catchments of the northern Murray–Darling Basin.

**Location:**

Northern Murray–Darling Basin, eastern Australia.

**Methods:**

A total of 122 individual plants from subpopulations located on rivers in four adjacent catchments were genotyped using 10 microsatellite markers. Microsatellite data were then analyzed using population genetic techniques to evaluate levels of gene flow and genetic structure and identify factors influencing dispersal.

**Results:**

Results suggest high levels of gene flow between lignum subpopulations of the northern Murray–Darling Basin. AMOVA revealed small but significant differences between subpopulations, and STRUCTURE analysis did not detect meaningful structure when sampling information was not provided. However, when sampling information was supplied using the LOCPRIOR model, three genetic clusters were identified. All Lower Balonne subpopulations were assigned to cluster 1 while a number of the other subpopulations showed mixed ancestry. Weak relationships were identified between pairwise genetic distance and geographic as well as river distance, although the *R* ^2^ value of the former was only half that of the latter.

**Main conclusions:**

Patterns of genetic variation suggest frequent long‐distance overland gene flow largely as a result of the movement of seeds via floodwater. Therefore, maintenance of natural variability in flow regime is key both to maintain conditions favorable to recruitment and to promote dispersal and gene flow across the landscape. However, given future climate change projections persistence may be more reliant on the species ability to endure long periods of drought between flood events.

## 1 INTRODUCTION

According to the latest estimates, drylands cover around 45% (~67 million km^2^) of the global land surface and are home to over 38% of the world's human population (Prăvălie, 2016; Reynolds et al., 2007). Unfortunately, they are also subject to widespread degradation with potential negative effects for more than 2.5 billion people. For this reason, increased understanding of the structure and functioning of these systems is important. The biophysical characteristics of dryland areas make them especially vulnerable to drivers of global change (Maestre, Salguero‐Gómez, & Quero, 2012). In particular, changes in rainfall and temperature that are associated with climate change have the potential for severe negative impacts (Maestre, Salguero‐Gómez, et al., 2012). Similarly, in the absence of rapid adaptation, riparian systems have been identified as particularly susceptible to the impacts of climate change (Capon et al., 2013). This is largely because of a long history of alteration by humans resulting in widespread degradation (Tockner & Stanford, 2002), as well as the close relationship between climate variables and important processes in these systems, most notably the relationship between rainfall and flow regime. This suggests that riparian ecosystems of economically and ecologically important dryland rivers, such as the Murray–Darling Basin in Australia (MDB), are doubly at risk of climate change impacts.

The maintenance of plant biodiversity has been identified as a factor of crucial importance for the resilience of dryland systems in the face of climate change and desertification (Maestre, Quero, et al., 2012). *Duma florulenta* (Meisn.) T.M. Shust (hereafter “lignum”) is a large diecious shrub that is a keystone species throughout the wetland ecosystems of Australian dryland rivers (James, Capon, & Quinn, 2015; Jensen, Walker, & Paton, 2006; Maher & Braithwaite, 1992). Despite its ecological importance, the mechanisms that allow this species to disperse and persist in such a variable and unpredictable landscape are poorly understood.

Rivers are considered to be important passageways for the dispersal of riparian plant species (Johansson, Nilsson, & Nilsson, 1996; Nilsson, Brown, Jansson, & Merritt, 2010). Several studies have used genetic markers to explore gene flow and dispersal in riverine plants with mixed results. These studies have found evidence for (Liu, Wang, & Huang, 2006; Love, Maggs, Murray, & Provana, 2013; Pollux, Luteijn, Van Groendael, & Ouborg, 2009) and against (Honnay, Jacquemyn, Nackaerts, Breyne, & van Looy, 2010; van der Meer & Jacquemyn, 2015; Ritland, 1989; Tero, Aspi, Siikamäki, Jäkäläniemi, & Tuomi, 2003) unidirectional hydrochoric dispersal of propagules and attempted to assess the applicability of a number of different models of gene flow and dispersal in riverine plant populations (Markwith & Scanlon, 2007; Pollux et al., 2009; Tero et al., 2003). This research has largely explored linear versions of traditional models such as classic metapopulation (Pollux et al., 2009) and stepping stone models (Markwith & Scanlon, 2007). However, while rivers can generally be considered linear at a reach scale, at catchment and basin scales, rivers are a hierarchical network of tributaries and main river channels (Fagan, 2002). Recent research regarding the genetics of riparian plant populations reflects this by investigating nonlinear patterns that occur within river networks (Cushman et al., 2014; Prentis & Mather, 2008; Wei, Meng, Bao, & Jiang, 2015).

The stream hierarchy model (SHM) was originally developed to describe patterns of genetic structure in desert fishes and describes a situation in which an organism is restricted to dispersal within the river corridor (Meffe & Vrijenhoek, 1988). Under this model, distance between populations along the river is the main factor that results in a hierarchical organization of genetic structure, where populations in different catchments have a higher level of differentiation than populations on different rivers within catchments, which, in turn, have a higher level of differentiation than populations on the same river (Hughes, Schmidt, & Finn, 2009; Meffe & Vrijenhoek, 1988). In organisms that are capable of overland dispersal, an isolation‐by‐distance pattern (IBD) (Wright, 1943) is most likely and if their ability to disperse is particularly efficient, among‐population genetic differentiation may be insignificant with populations effectively acting as a single panmictic unit (Hughes et al., 2009). At the other end of the spectrum, the Death Valley model (DVM) describes a situation in which no current gene flow exists between populations resulting in high genetic differentiation between populations and low variation within populations (Hughes et al., 2009; Meffe & Vrijenhoek, 1988).

This paper utilizes microsatellite markers to explore gene flow, dispersal, and genetic structure in a keystone floodplain plant species that is widely distributed throughout the wetlands, floodplains, and riverbanks of Australia's dryland river systems. We hypothesized that lignum populations in the northern MDB would resemble those depicted by the SHM because this species exhibits numerous characteristics conducive to hydrochory (e.g., buoyant seeds, a riverine distribution, and the ability to reproduce from stem fragments broken off in floodwaters). In addition, we predicted that genetic diversity would increase downstream as a result of predominantly downstream dispersal.

## 2 METHODS

### 2.1 Study area

**Figure 1:**
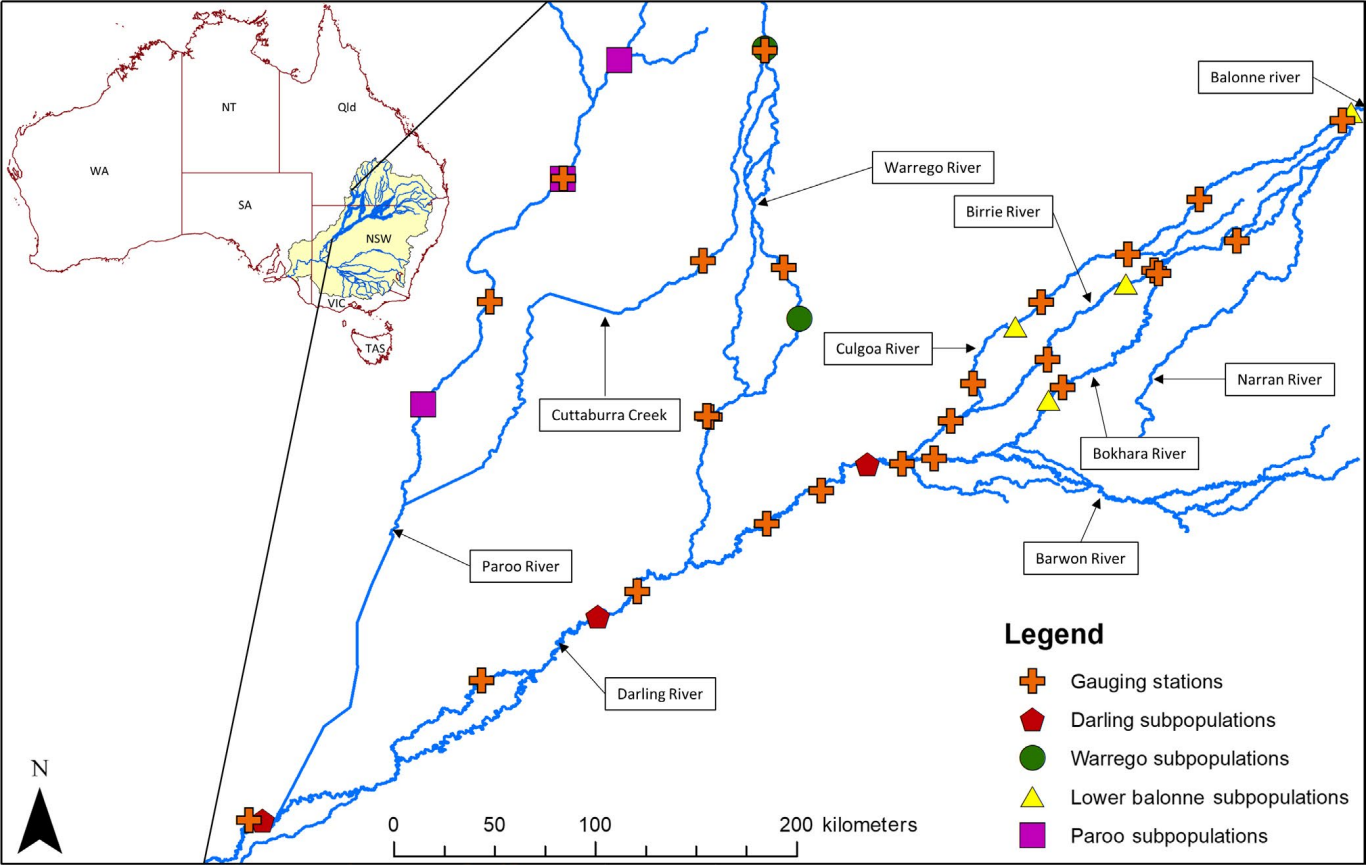
Study area showing lignum (*Duma florulenta*) subpopulations and flow gauging stations. The Murray–Darling basin is represented by the yellow‐shaded area of the insert, and gauging stations are numbered according to IDs in Table 2. Flow in the northern basin is in a southwesterly direction while flow in the southern basin is in a westerly direction with flow draining into the Southern Ocean via the Murray Mouth in South Australia during high flow periods

The MDB is the largest river system in Australia draining over 14 percent of the continent and consisting of a network of approximately 77,000 km of river channel (Figure 1). Throughout the MDB, there are over 25,000 wetlands, 16 of which are listed under the Ramsar Convention on wetlands of international importance (Australian Government, 2018). Wetlands of the MDB host significant bird breeding events that are integral to the survival of over 98 species of colonial and migratory waterbirds that have been recorded in the region. This importance is demonstrated by several areas being subject to international bird agreements such as the Japan–Australia Migratory Bird Agreement and the Republic of Korea–Australia Migratory Bird Agreement.

Average annual rainfall in the MDB ranges from over 1,000 mm in the areas of the Great Dividing Range in the south and east of the catchment and to as low as 100 mm in the western margins. Annual discharge for the basin is generally low but highly variable with an average of approximately 32,500 gigaliters (GL). The northern MDB includes the Darling River and all catchments draining into it while the southern MDB includes the Murrumbidgee, the Murray, and all catchments these rivers drain. Flows in the southern part of the MDB are relatively less variable and more predictable as result of higher levels of regulation and a more seasonal high flow season stemming from winter rainfall and spring snowmelt. Flows in the northern MDB are more variable with the Darling River being ranked among the most hydrologically variable and unpredictable rivers in the world (Puckridge, Sheldon, Walker, & Boulton, 1998). Despite occupying 60% of its total area, the northern part of the MDB only accounts for 32% of the total flows. This study focused on lignum subpopulations located in four of the northern catchments of the MDB: the Warrego, the Paroo, the Darling, and the Condamine–Balonne. Given population limits have not been clearly established in the species or study area, this paper uses the term subpopulation to describe the group of individual plants sampled at each site.

### 2.2 Study species

Lignum is a woody perennial shrub with numerous intertwining branches forming essentially rounded clumps under favorable conditions. During dry periods, lignum adopts a brown leafless form that can appear void of life; however, the species is capable of responding quickly to rainfall and flooding to produce new green stems, leaves, and flowers (Craig, Walker, & Boulton, 1991). Flooding is essential to the survival of lignum populations, and the species generally occupies habitats that have flood frequencies ranging from once per year to once every ten years (Roberts & Marston, 2011). Dormancy during long periods of drought followed by regeneration from rootstocks is critical for the persistence of lignum in these variable environments. It is not known how long lignum can survive in poor condition prior to dormancy; however, four years is the maximum observed period for which an individual has remained dormant and still successfully regenerated (Freestone et al., 2017). Lignum can reproduce sexually through seed and asexually through branch layering, rhizomes, and stem fragments that are broken off and distributed by floodwaters. Reproduction is more opportunistic than seasonal with flowering occurring in response to rainfall and flooding. This process is rapid and the time from anthesis to the production of viable seed can be as little as 14 days (Chong & Walker, 2005). Seeds remain buoyant for up to 49 days and germinate readily given moisture and fluctuating temperatures (Chong & Walker, 2005; Higgisson, Briggs, & Dyer, 2018). A lack of viable seeds in soil samples and the absence of lignum seedlings in soil germination experiments (Capon & Reid, 2016; Dawson et al., 2017; Reid, Reid, & Thoms, 2016) indicate that seeds do not remain viable for long periods either on the plant or in the soil, suggesting the species is not reliant on a dormant soil seed bank (Chong & Walker, 2005). Seedlings have been found to be tolerant of both flooding and drying: responding to inundation via reduced growth and drying through plastically reducing leaf area ratios (Capon, James, Williams, & Quinn, 2009).

### 2.3 Field sampling of DNA extraction

Samples were collected from 13 sites located on seven different rivers of the northern MDB (Figure 1). Sites were selected in areas considered close enough to the river channel for dispersal of seeds by the river to be possible. Sites were selected at similar proximities both between rivers and within rivers to facilitate the identification of dispersal via hydrochory and/or overland dispersal. The distribution of riverine vegetation in Australian dryland rivers is dictated by complex relationships between the hydrological regime and the physical template of the floodplain and river channel (Casanova & Brock, 2000; Scown, Thoms, & De Jager, 2016). This relationship creates a mosaic of different habitat patches that are heterogeneous in both space and time. As a result, lignum occurs in discrete groups of individuals that are dotted across the floodplain, along riverbanks, within river channels, and adjacent to or within ephemeral lakes or water bodies. For the current study, lignum was distributed in somewhat isolated patches across the floodplain at Darling River sites with no plants growing along the riverbank. The subpopulation at Trilby was particularly small and isolated consisting of ≤~50 individuals. The occurrence of the species was comparatively more continuous and not restricted to the floodplain in the Warrego, Paroo, and Lower Balonne subpopulations with majority of those sampled occurring either on the riverbank or within the river channel. Only two subpopulations were not located on the riverbank for these rivers. The subpopulation for the Birrie River was located within a depression that extended approximately 500 m perpendicular to the riverbank, and samples were collected from individuals located along the extent of this depression. Culgoa samples were collected from a small isolated subpopulation located on a small tributary about 500 m from the main channel.

Resource limitations and the condition of plants within each subpopulation meant that the number of individuals we were able to sample was limited to a total of 122 lignum tissue samples from randomly selected adult individuals across the 13 sites (3–15 at each site). Where possible, samples were collected at a distance of at least 20 m from each other in order to avoid the collection of clonal ramets with identical genotypes at a local scale. For each sample, 20 mg of silica‐dried leaf or stem tissue, depending on availability at time of sampling, was ground mechanically using a Mixer Mill MM 301 (Retsch GmbH & Co.) at 30 Hz for 2 min (leaf) to 4 min (stem). Genomic DNA was isolated using a Bioline ISOLATE II Plant DNA Kit (Bioline) according to the manufacturer's protocol. Following isolation, purity and concentration of DNA samples were determined using a NanoDrop 8000 Spectrophotometer (Thermo Fisher Scientific).

### 2.4 Genotyping

Samples were genotyped at 12 microsatellite loci reported for lignum by Murray, Reid, and Wu (2018) (Genbank accession numbers KX762272–KX762283). To reduce the costs by multiplexing, PCR products were labeled using M13 universal primers as outlined in Sheulke (2000). Total PCR volumes were 15 µl consisting of 160 nM reverse primer and fluorescently tagged M13 universal primer sequence (TGT AAA ACG ACG GCC AGT), 40 nM of the forward primer with M13 tail, 1× Bioline MyTaq reaction buffer, 0.75 U Bioline MyTaq DNA polymerase, and 30 ng DNA template. A touchdown PCR program was used, which consisted of an initial denaturation step of 5 min at 94°C followed by three cycles of denaturation for 30 s at 94°C, annealing for 45 s at 60°C, and elongation for 45 s at 72°C. This step was repeated for three cycles with annealing temperatures of 57°C and 54°C and for 30 cycles at an annealing temperature of 52°C. The last step was a final elongation at 72°C for 10 min. Multiplex microsatellite analysis was performed using a multiplex genotyping method where PCR products were amplified in simplex and then mixed before loading into the same electrophoresis gel channel, that is, sequencer capillary (Vieira, Santini, Diniz, & Munhoz, 2016). PCR products were analyzed with applied Biosystems GeneScan LIZ‐500 on a 3730 Genetic Analyzer. Alleles were scored using GeneMapper v 4.0, and 18 bp was subtracted from total fragment sizes to account for the effect of adding the M13 primer tail to locus‐specific forward primers.

### 2.5 Hydrological data

Daily discharge data (ML/day) were retrieved for 25 gauging stations in the study area from the Queensland Water Monitoring Information Portal (https://water-monitoring.information.qld.gov.au/) and the New South Wales Water Information website (http://waterinfo.nsw.gov.au/) (Figure 1). For each gauging station, the proportion of days where no flow occurred as well as the Autumn/Spring frequency of flow events with magnitudes above the 75th and 90th percentile were calculated using the Hydrostats package in R (Bond, 2015; R Core Team, 2015). Given the ephemeral nature of rivers in this area, days with flows of zero were ignored when calculating flow percentiles to avoid negatively skewing threshold calculations. Figures for gauging stations located between all pairwise combinations of subpopulations were averaged in order to obtain a single figure for each pairwise comparison. Many of the pairwise combinations of subpopulations do not require upstream movement for seed dispersal via water between them. However, for subpopulations located in different river catchments dispersal via hydrochory generally requires movement through an upstream segment. This is the case for all pairwise combinations that involve subpopulations located on different rivers except those involving the Darling as all the rivers in the northern MDB eventually flow into this river. Dispersal via hydrochory generally only occurs in a downstream direction. To take this into account when calculating flow variables, in situations where connection between subpopulations required movement through a stream segment against the direction of flow, all gauging stations within that segment were set to zero for frequency measures and one (100%) for the proportion of no flow days For consistency, the westernmost segments were always considered to be the upstream segments.

### 2.6 Statistical analysis

Given dry conditions during field sampling, samples from which DNA of sufficient quality could be extracted were only obtained from three individuals at both the Culgoa and Tinnenburra subpopulations. As a result, these two subpopulations were excluded from all data analysis except the cluster analysis. Polymorphic information content (PIC) was estimated using CERVUS (Kalinowski, Taper, & Marshall, 2007), and the presence of linkage disequilibrium and deviations from Hardy–Weinberg equilibrium (HWE) were estimated using GenePop 4.4.3 (Rousset, 2008). Markers with a PIC > 0.5 are considered to be highly informative, markers with PIC > 0.25 are considered to be moderately informative while markers with PIC < 0.25 are considered to have low information content (Langen, Schwarzer, Kullman, Bakker, & Thünken, 2011). Inbreeding coefficients were calculated in FSTAT (Goudet, 2001), and GenAlEx 6.503 (Peakall & Smouse, 2006, 2012) was used to estimate the number of alleles, observed and expected heterozygosities, and probability of identity (PI). Calculations for PI included both a regular PI equation that does not take into account the possibility of related individuals being sampled and a more stringent equation that accounts for the sampling of relatives, PI_sibs_. MicroChecker 2.2.3 (van Oosterhaut, Hutchinson, Willis, & Shipley, 2004) was used to detect the presence of null alleles and genotype scoring errors.

Genetic differentiation between subpopulations was explored using the *F*_ST _measure of population subdivision. Genetic structure at subpopulation and river system levels was investigated using a hierarchical analysis of molecular variance (AMOVA) (Excoffier, Smouse, & Quattro, 1992) conducted in GenAlEx 6.503. Patterns of genetic differentiation were initially explored through principal coordinates analysis (PCoA). In order to determine the importance of the river system in facilitating gene flow, the relationship between genetic differentiation and a number of landscape variables was examined. Independent variables were designed to allow differentiation between possible dispersal vectors (i.e., wind, water, or animals) and included natural log‐transformed pairwise geographic distance (Euclidean distance between subpopulations), natural log‐transformed pairwise river distance (distance along river network between subpopulations), a measure of wind (i.e., prevailing wind direction in comparison with angle between two sites), proportion of no flow days, and the frequency of 75th and 90th percentile floods occurring in Autumn and Spring months. It has been suggested that successful recruitment in lignum is reliant on a flood event to promote flowering and seed set followed by a second to promote germination (Roberts & Marston, 2011). Germination events are more likely to be successful if this second round of flooding occurs in Autumn or Spring as temperatures likely to inhibit germination are less common during these periods. This was the reasoning behind the seasonality of the flood frequency variables. The relationship between each of these variables and genetic distance as represented by pairwise *F*_ST _values was tested using Mantel tests with significance based on 9,999 permutations both individually using GenAlEx 6.503 (Peakall & Smouse, 2006, 2012) and in combination via a partial Mantel test carried out using the package Phytools in R. Significant autocorrelation was present between the two flow frequency variables so only the 90th percentile flood frequency variable was used in the partial Mantel test.

Finally, Bayesian cluster analysis was implemented, in STRUCTURE version 2.3.4 (Pritchard, Stephens, & Donnelly, 2000), in order to identify whether individuals could be grouped into K panmictic genetic clusters. The analysis was performed using the admixture model with a burn‐in period of 100,000 iterations followed by 500,000 Markov chain Monte Carlo (MCMC) iterations. K was set at 1–13, and 10 independent runs were carried out for each level of K. Analysis was performed both with no prior information and using the sampling location as prior information (LOCPRIOR model). Further interpretation of the results obtained from STRUCTURE was carried out using STRUCTURE HARVESTER (Earl & vonHoldt, 2012) which utilizes Evanno's Δ*K* method (Evanno, Regnaut, & Goudet, 2005) to identify the optimal number of *K* population clusters. The Greedy algorithm option in CLUMPP version 1.1.2 (Jakobsson & Rosenberg, 2007) was selected to identify the optimal alignment of the 10 independent runs. The resulting clusters were visualized using Distruct version 1.1 (Rosenberg, 2004).

## 3 RESULTS

### 3.1 Microsatellite characteristics and genetic diversity

Two of the microsatellites (Df 45 KX762272 and Df 88 KX762282) failed to amplify with 28% and 20% of individuals, respectively, so these data were not included in further analysis. Null alleles were detected at 3 (Wilcannia, Bokhara, Weir), 2 (Wanaaring, Trilby), 2 (Lagoon, Balonne), and 1 (Wanaaring) subpopulation for loci Df 80, Df 78, Df 40, and Df 3, respectively. At locus Df 87, however, null alleles were detected at all but four subpopulations (Wilcannia, Culgoa, Balonne, and Tinnenburra). For this reason, Df 87 was excluded from further analysis while Df 80, Df 78, Df 40, and Df 3 were retained in order to maintain statistical robustness, given that null alleles were only detected in a small portion of subpopulations for these loci. A total of 122 individuals from 13 subpopulations were scored at the remaining nine microsatellite loci revealing 81 alleles, with alleles per locus ranging from 2 to 18. Linkage disequilibrium was not significant for all 36 pairwise locus combinations across all subpopulations. Exact tests indicated significant deviation from HWE for 12 of 117 locus–subpopulation combinations at an alpha of 0.05. This included 2 (Trilby, Corni‐Paroo) at locus Df 100, 3 (Trilby, Weir, Eulo) at locus Df 3, 3 (Lagoon, Balonne, Enngonia) at locus Df 40, 2 (Trilby, Wanaaring) at locus Df 78, and 2 (Wilcannia, Bokhara) at locus Df 80. Following sequential Bonferroni correction (Rice, 1989), 1 combination remained significant, Df 3 at Trilby (Darling). The PIC of markers across all individuals spanned from low to high with values ranging between 0.069 and 0.840 and an overall mean of 0.438. Observed heterozygosities ranged from 0.200 at Corni‐Paroo to 0.429 at the Birrie River site, and expected heterozygosities ranged from 0.391 at Corni‐Paroo to 0.593 at the Tinnenburra site on the Warrego. No downstream increase in levels of expected heterozygosities or number of alleles per locus was observed (Table 1). Observed heterozygosities were consistently lower than expected, and all but three subpopulations (Wilcannia, Birrie, and Eulo) showed significant inbreeding coefficients (*F*_is_) (*α* = 0.05). However, following Bonferroni correction significant *F*_is_ was restricted to Trilby, Culgoa, and Tinnenburra subpopulations (Table 1). Cumulative PI values were less than 0.0013 for all subpopulations with an overall value of 0.00016 while PI_sibs _values were less than 0.05 for all subpopulations with an overall value of 0.0146 meaning that the suite of markers could be confidently used for clonal identification. However, no multilocus genotype matches were found between any of the lignum individuals.

**Table 1:** Characteristics of the 12 lignum (*Duma florulenta*) subpopulations and marker suite

River system	Pop	*N*	Na	*H*_o_	*H*_E_	PI	PI_sibs_	*F*_is_
Darling	Lagoon	14	4.1	0.379	0.483	1.8E−05	0.0086	0.222***
	Trilby	10	3.2	0.351	0.519	3.0E−05	0.0067	**0.339** ****
	Wilcannia	10	3.4	0.378	0.440	7.0E−05	0.0147	0.149
Lower Balonne	Balonne	15	3.8	0.391	0.441	3.6E−05	0.01287	0.116*
	Birrie	14	3.9	0.437	0.446	4.7E−05	0.0127	0.022
	Culgoa	3	2.3	0.222	0.519	1.0E−04	0.0132	**0.625** ***
	Bokhara	14	3.2	0.336	0.408	3.8E−04	0.0201	0.182*
Warrego	Weir	9	3.8	0.362	0.464	1.3E−05	0.0104	0.232**
	Tinnenburra	3	2.4	0.222	0.556	4.2E−05	0.0092	**0.652** ****
	Enngonia	10	3.0	0.342	0.444	1.1E−04	0.0151	0.240**
Paroo	Eulo	5	2.6	0.439	0.479	1.3E−04	0.0147	0.092
	Corni‐Paroo	5	2.0	0.200	0.343	1.3E−03	0.0453	0.446**
	Wanaaring	10	4.1	0.433	0.512	6.7E−06	0.0064	0.161*
Overall		122	3.22	0.346	0.466	1.6E−04	0.0146	**–**

Note

Pop = subpopulation (subpopulations are presented in order from upstream to downstream within each river system), *N* = number of individuals sampled, Na = mean number of alleles per locus, *H*_O_ = observed heterozygosity, *H*_E_ = expected heterozygosity, PI = probability of identity, PI_sibs_ = probability of identity taking into account related individuals, *F*_is_ = inbreeding coefficient.

Bolded *F*s are significantly different from 0 following Bonferroni correction.

* *p* < 0.05.

** *p* ≤ 0.01.

*** *p* ≤ 0.001

**** *p* ≤ 0.0001.

### 3.2 Hydrological data

In general, the Darling River was the least variable of the rivers in terms of flow with gauging stations recording the lowest proportions of no flow days and also comparatively low frequencies of 90th and 75th percentile flow events (Table 2). The highest proportions of no flow days were recorded at the Bokhara River, Birrie River, Warrego River, and Cuttaburra Creek gauging stations while the highest frequencies of 75th and 90th percentile floods were recorded at Paroo river gauging stations (Table 2).

**Table 2:** Flow data at 25 gauging stations within the Murray–Darling Basin. Includes Autumn/Spring frequency of 75th and 90th percentile flood events and the proportion of days with no flow over the entire record

ID	Gauging station	75th Autumn/Spring	90th Autumn/Spring	Duration no flow	Length of data record (years)
1	Barwon @ Beemery	0.941	0.412	0.040	17
2	Birrie @ Goodooga	0.885	0.462	0.654	53
3	Birrie @ Talawanta	0.712	0.269	0.666	53
4	Bokhara @ Hebel	0.981	0.442	0.562	52
5	Bokhara @ Goodooga Weir	1.000	0.500	0.399	8
6	Bokhara @ Bokhara	0.681	0.375	0.598	73
7	Culgoa @ Whyenbah	1.686	0.863	0.298	51
8	Culgoa @ Woolerbilla	1.000	0.423	0.313	52
9	Culgoa @ Brenda	1.393	0.732	0.387	57
10	Culgoa @ Weilmoringle	1.192	0.558	0.450	53
11	Culgoa @ Collerina US	1.137	0.510	0.325	53
12	Culgoa @ Collerina DS	1.306	0.500	0.312	73
13	Cuttaburra @ Turra	1.087	0.435	0.624	24
14	Darling @ Warraweena	1.063	0.438	0.023	18
15	Darling @ Bourke	0.628	0.347	0.052	122
16	Darling @ Weir DS	1.071	0.500	0.015	15
17	Darling @ Louth	0.685	0.351	0.207	112
18	Darling @ Tilpa	1.000	0.571	0.054	22
19	Darling @ Wilcannia	1.010	0.437	0.065	104
20	Paroo @ Caiwarro	2.204	1.102	0.389	49
21	Paroo @ Willara	1.732	0.976	0.365	41
22	Warrego @ Cunnamulla	0.960	0.520	0.565	25
23	Warrego @ Barringun	1.087	0.478	0.506	23
24	Warrego @ Fords Bridge Bywash	1.170	0.543	0.459	96
25	Warrego @ Fords Bridge Main Channel	0.727	0.273	0.653	44

Note

Gauging stations are presented from upstream to downstream for each river, and IDs correspond to those presented in Figure 1. Final year of data records was 2016.

### 3.3 Genetic structure

**Figure 2:**
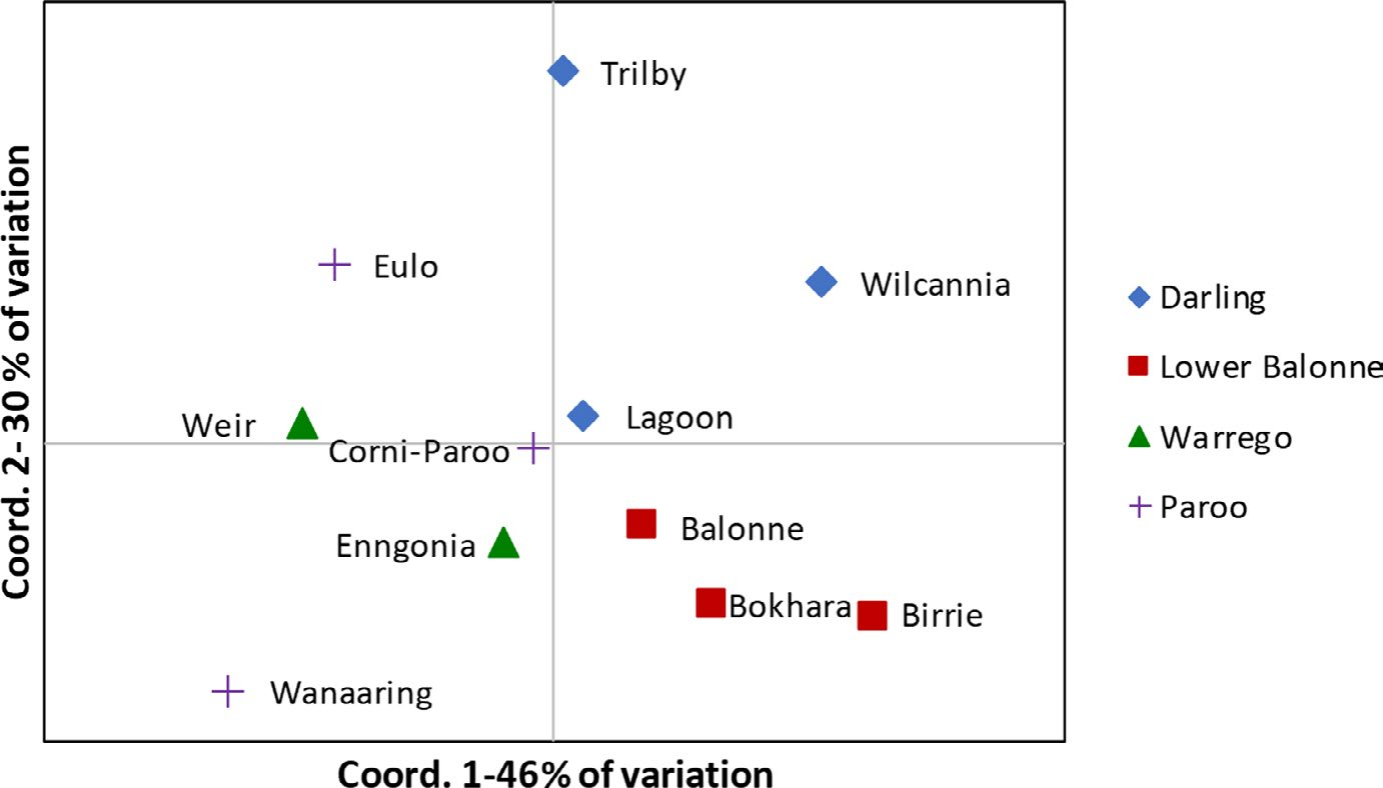
Principal coordinates analysis of pairwise genetic differentiation between 11 lignum (*Duma florulenta*) subpopulations in the northern Murray–Darling Basin based on pairwise *F*_ST_ measures

**Figure 3:**
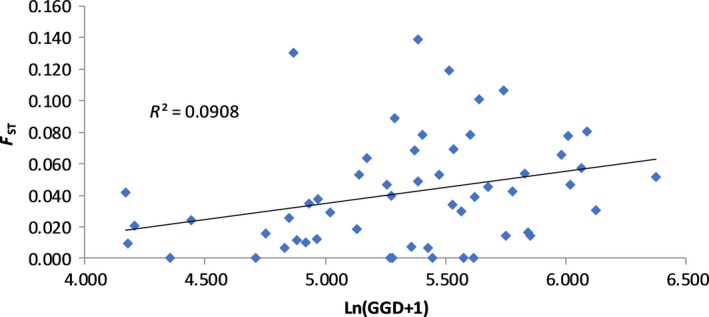
Mantel tests showing the relationship between pairwise genetic distance and natural log‐transformed geographic distance in 11 subpopulations of lignum (*Duma florulenta*) in the northern Murray–Darling Basin

**Figure 4:**
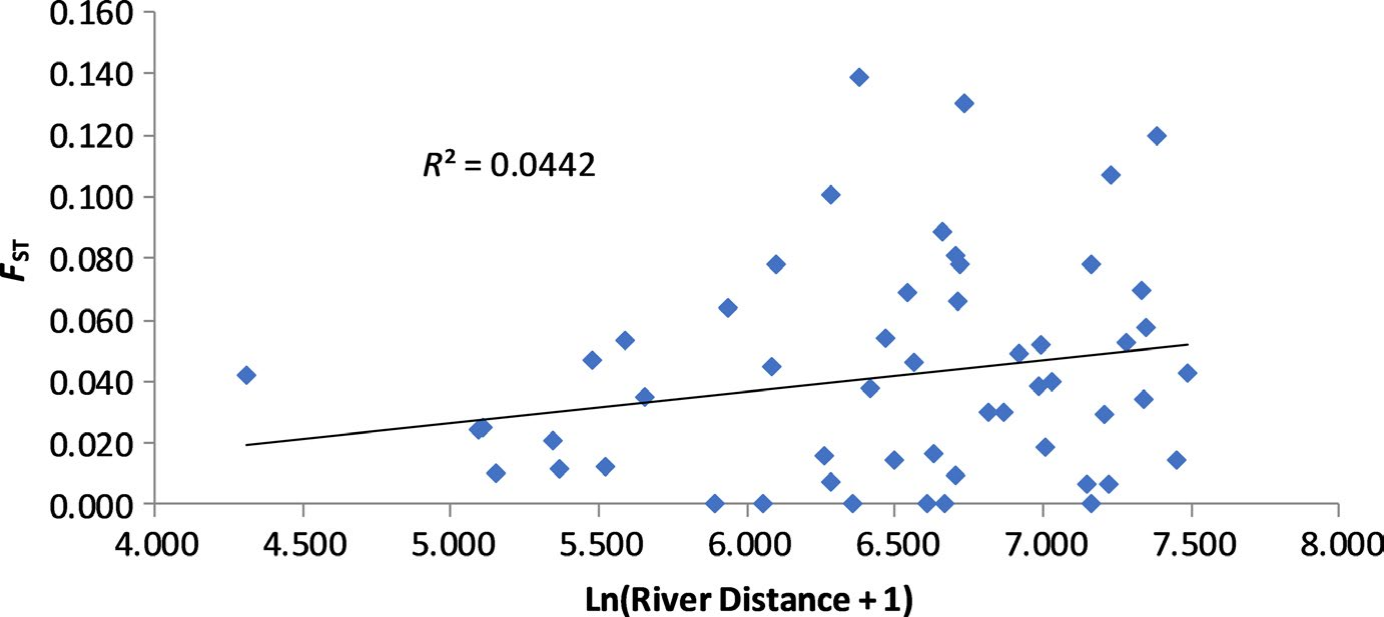
Mantel tests showing the relationship between pairwise genetic distance and natural log‐transformed river distance in 11 subpopulations of lignum (*Duma florulenta*) in the northern Murray–Darling Basin

Hierarchical AMOVA indicated that the majority (~95%) of variation in lignum could be attributed to variation within rather than among subpopulations. Of the variation that could be attributed to differences among subpopulations, the majority was the result of differences between subpopulations within river systems (*F*_SR_ = 0.023, *p* ≤ 0.0001) (Table 3). However, differences among river systems (*F*_RT_ = 0.021, *p* ≤ 0.0007) and between subpopulations overall were also significant (*F*_ST_ = 0.044, *p* ≤ 0.0001) (Table 3). PCoA carried out on genetic differentiation matrices created by the AMOVA analysis showed grouping of Lower Balonne subpopulations, in particular, with Darling River subpopulations also grouping together but with more variation between subpopulations particularly the Trilby subpopulation which was somewhat separated from the other two (Figure 2). The Paroo and Warrego river subpopulations were grouped together on the left of the plot, again with more variation between subpopulations than observed for Lower Balonne subpopulations, particularly among the Paroo River subpopulations. The percentage of variation explained by the first two axes was equal to around 76%. Overall, the genetic differentiation between subpopulations was low to moderate with significance tests showing that the majority of differences were between Trilby, Wilcannia, and Wanaaring, and each other as well as other subpopulations (Table 4), and Birrie and Bokhara and other subpopulations (Table 4). Mantel tests revealed a significant positive relationship between pairwise *F*_ST _values and natural log‐transformed straight‐line geographic distance with an *R* ^2^ value of 0.091 (*R*_XY_ = 0.301, *p* < 0.036) (Figure 3). The relationship between pairwise genetic distance and natural log‐transformed river distance was also positive and significant; however, the amount of variation explained by river distance was about half that of geographic distance with an *R* ^2^ of 0.044 (*R*_XY_ = 0.210, *p* < 0.044) (Figure 4). This indicates that pairwise genetic differentiation increases with distance between subpopulations both along the river and according to straight‐line Euclidean distance, the latter having a greater effect than the former. Neither the wind (*R*_XY_ = −0.009, *p* = 0.440) nor any of the flow variables (90th percentile: *R*_XY_ = −0.054, *p* = 0.376; 75th percentile: *R*_XY_ = −0.098, *p* = 0.237; proportion of no flow days: *R*_XY_ = −0.061, *p* = 0.353) exhibited significant relationships with pairwise genetic distance as measured by *F*_ST_. Partial Mantel analysis used to identify relationships that might arise from the effect of interactions between variables was also not significant when all but the 75th percentile independent variable were analyzed in unison (*R*_XY_ = 0.311, *p* = 0.684).

**Table 3:** Results of hierarchical AMOVA for 11 subpopulations of lignum (*Duma florulenta*) based on nine nuclear microsatellite loci

Source of variation	*df*	Sum of squares	Variance components	Percentage of variation
*F*_ST_				
Among river systems	3	19.9	6.6	2.13
Among subpopulations within river systems	7	27.4	3.9	2.25
Among individuals within subpopulations	105	297.0	2.8	27.15
Within individuals	116	183.0	1.6	68.47
Total	231	527.3		

*F* statistics	Value	*p* Value
*F*_RT_	0.021	0.0001
*F*_SR_	0.023	0.0007
*F*_ST_	0.044	0.0001
*F*_IS_	0.284	0.0001
*F*_IT_	0.315	0.0001

Note

*p* Values based on 9,999 random permutations.

**Table 4:** Pairwise genetic differentiation matrix and corresponding geographic distance matrix

Lagoon	Wilcannia	Trilby	Bokhara	Birrie	Balonne	Enngonia	Weir	Eulo	Corni‐Paroo	Wanaaring	
	313.576	137.874*	840.87*	142.610	273.534	76.828	210.954	227.032	193.236	194.166*	Lagoon
0.0144		**175.801** ***	395.843**	456.060	586.761**	338.502**	440.407**	407.115**	343.261	**217.643** ****	Wilcannia
0.0346*	**0.0638** ***		**220.851** ***	**280.264** ****	410.972**	169.928**	290.624*	275.257	216.831*	**129.240** ****	Trilby
0.0242*	0.0657**	**0.0781** ***		66.303	194.929	114.921	214.112**	251.587*	237.774*	**270.367** ***	Bokhara
0.0121	0.0302	**0.1007** ****	0.0205		130.974	143.002*	**196.959** ***	**247.787** ***	251.012	**310.445** ****	Birrie
0.0000	0.0517**	0.0465**	0.0000	0.0115		262.443	259.747*	322.518*	347.067	429.698**	Balonne
0.0000	0.0540**	0.0531**	0.0160	0.0379*	0.0000		136.070	150.840	124.496	168.215	Enngonia
0.0071	0.0808**	0.0451**	0.0687**	**0.0888** ***	0.0298*	0.0100		64.225	110.026	231.216	Weir
0.0069	0.0779**	0.0387	0.0694*	**0.1195** ***	0.0428*	0.0294	0.0092		63.865	190.634	Eulo
0.0000	0.0167	0.0487*	0.0528*	0.343	0.0145	0.0064	0.0000	0.0418		126.963	Corni‐Paroo
0.0400*	**0.1388** ****	**0.1302** ****	**0.0782** ***	**0.1067** ****	0.0576**	0.0184	0.0000	0.0468	0.0253		Wanaaring

Note

*F*_ST_ values are below the diagonal geographic distance values in kilometers are above the diagonal.

Results that remain statistically significant after sequential Bonferroni correction are bolded.

* *p* < 0.05.

** *p* ≤ 0.01.

*** *p* ≤ 0.001.

**** *p* ≤ 0.0001.

**Figure 5:**
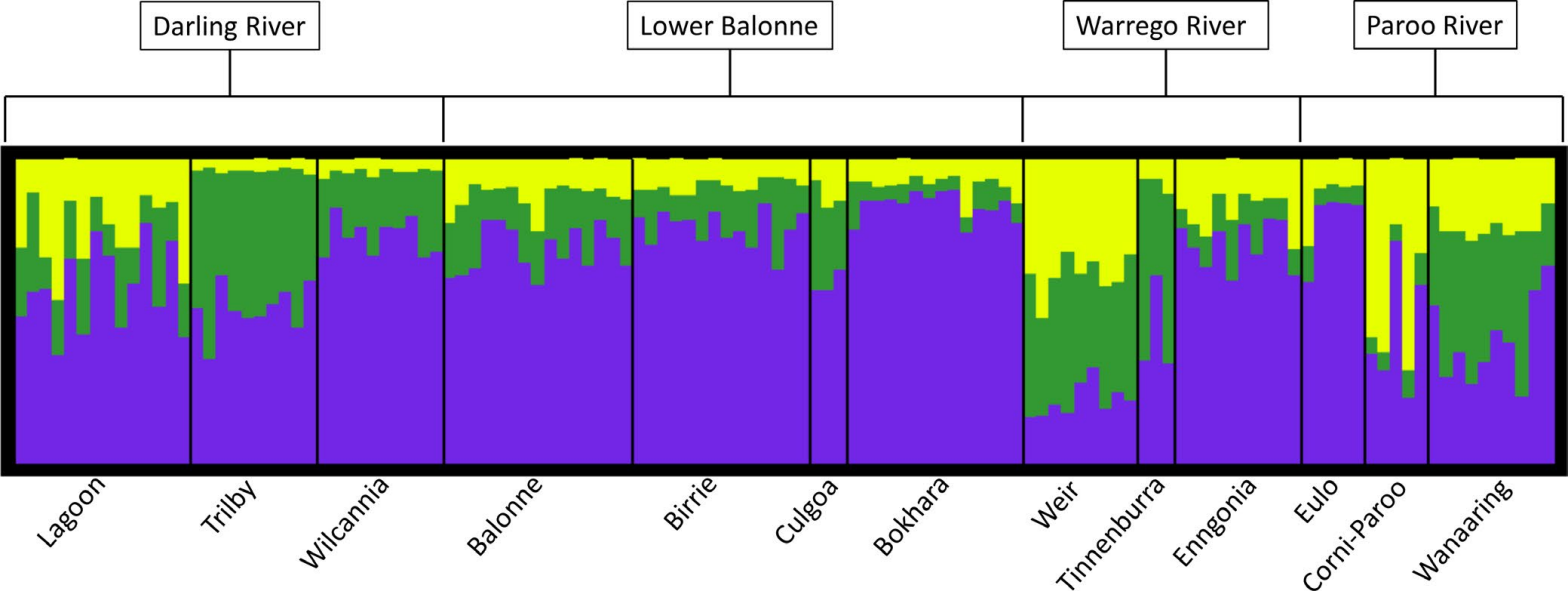
Partitioning of *K* = 3 genetic clusters based on Bayesian clustering using the LOCPRIOR model in STRUCTURE. Bottom axis indicates subpopulations of lignum (*Duma florulenta*) in the northern Murray–Darling Basin. Top axis indicates river system. Each bar represents the proportion of each individual plant assigned to each of the two genetic clusters. Purple = cluster 1; green = cluster 2, and yellow = cluster 3

Evanno's Δ*K* method applied to the results of STRUCTURE analysis without LOCPRIOR information identified *K* = 4 genetic clusters as the optimal solution, mixed ancestry was apparent for all individuals, and no meaningful grouping was detected, suggesting all 13 subpopulations are a part of a single panmictic population. The Evanno method is incapable of selecting *K* = 1 even if this is the true scenario (Evanno et al., 2005) and examination of the posterior probabilities of *K* found that *K* = 1 had the highest value with steady decrease for subsequent levels of *K*, further suggesting that a single panmictic population was the true situation. However, in situations where datasets provide limited information on population structure, either because of limited number of markers or sampled individuals, the program STRUCTURE provides the LOCPRIOR option which allows information on sampling locations to be used to achieve better results when determining the ancestry of individuals (Hubisz, Falush, Stephens, & Pritchard, 2009). When the LOCPRIOR option was implemented using subpopulation IDs as prior information, the Δ*K* approach suggested an optimal solution of *K* = 2 genetic clusters. On this occasion, the posterior probability of *K* = 2 was higher than *K* = 1, and although *K* = 3 was higher, the difference was not substantial, a situation in which the authors of the program suggest the selection of the smaller value (Pritchard, Wen, & Falush, 2010). The tuning parameter, *r*, measures the usefulness of the sampling location information (Hubisz et al., 2009). Small values of *r* near 1 or <1 indicate that sampling locations are informative while values substantially greater than 1 indicate either a lack of population structure or that structure is independent of locations. The mean *r* across the 10 runs for *K* = 2 was 1.618 while the *r* for *K* = 3 was 1.056, suggesting that the sampling information was of greater use in the model *K* = 3. For this reason, *K* = 3 was selected as the optimal number of clusters. All of the Lower Balonne subpopulations were assigned to cluster 1 excluding the Culgoa subpopulation which only consisted of three individuals and was not included in other analyses of genetic structure (Figure 5). Other subpopulations that could be assigned to cluster 1 included Wilcannia, Enngonia, and Eulo. The other subpopulations showed mixed ancestry with cluster 3 becoming more prominent in the Lagoon and Corni‐Paroo subpopulations, clusters 2 and 3 becoming more prominent in Weir and Wanaaring subpopulations, and cluster 2 becoming more prominent in Trilby and Tinnenburra subpopulations (Figure 5).

## 4 DISCUSSION

### 4.1 Genetic diversity

Overall levels of within‐ and among‐subpopulation genetic diversity in this study were low, and patterns of downstream increase in genetic diversity were not evident. Expected heterozygosity provides a valuable and regularly used measure of genetic diversity (Rosenberg & Kang, 2015). Expected heterozygosity levels (0.343 to 0.556) in this study were lower than expected for long‐lived perennial, widespread, and outcrossing plant species (~0.65) (Nybom, 2004) as well as other Australian riparian shrub (0.69) (Hevroy, Moody, & Krauss, 2017) and tree species (~0.8) (Butcher, McDonald, & Bell, 2009). No clear patterns of downstream increase in expected heterozygosity or the number of alleles per locus were observed (Table 1), suggesting no evidence for the unidirectional dispersal hypothesis as predicted under dominant downstream dispersal of seeds by hydrochory. This is not unusual for plants inhabiting riverine systems with numerous studies failing to detect evidence for unidirectional downstream dispersal (Honnay et al., 2010; Markwith & Scanlon, 2007; Ritland, 1989). As previously mentioned, lignum can reproduce asexually via fragments of stem that break off the mother plant, are dispersed, and subsequently take root at another location (Roberts & Marston, 2011). The movement of these stem fragments by flood water could result in long‐distance vegetative dispersal and decreased levels of genetic variation both within and among subpopulations. However, no multilocus genotype matches were found either within subpopulations or among them in this study, so this could not be confirmed.

Homozygote excess at the majority of subpopulations resulted in a number of deviations from HWE as evidenced by positive *F*_is_ at all subpopulations. This is not a surprising result given that lignum is capable of regular reproduction via several modes of asexual reproduction (Roberts & Marston, 2011). Following Bonferroni correction, *F*_is _values for Culgoa, Tinnenburra, and Trilby remained significant. Culgoa and Tinnenburra were the two subpopulations with the highest *F*_is _and the smallest number of sampled individuals. The small sample size at these sites was clearly affecting the ability to pick up the full scope of heterozygotes at these subpopulations with *F*_is _values exceeding 0.6; however, these subpopulations were omitted from the majority of genetic analyses. Conditions that promote sexual recruitment via seed are rare within the dryland river ecosystems that this species occur, and asexual reproduction is likely to be common during periods that may be favorable to growth but not conducive to seedling survival. Trilby on the Darling was a small isolated subpopulation located some distance from the river channel, so elevated levels of inbreeding might be expected as a result of increased asexual reproduction and lower levels of gene flow from other subpopulations. The detection of possible null alleles suggests that homozygote excess responsible for significant inbreeding coefficients and deviations from HWE could be the result of a combination of biological factors and artifacts of genotyping and sampling design (i.e., relatively small samples sizes). However, reproductive characteristics of the species would suggest that homozygote excess as observed here could be expected in the sampled subpopulations. In addition, although null alleles were detected they were only detected at a small proportion of the subpopulations for any one marker.

### 4.2 Genetic structure and dispersal

Similar to other genetic studies of long‐lived perennial tree and shrub species, most of the genetic variation in lignum was within rather than among subpopulations (Butcher et al., 2009; Hevroy et al., 2017; Wei, Meng, & Jiang, 2013; Table 3). Variation between subpopulations was small but highly significant, and STRUCTURE was unable to cluster individuals without the provision of sample location information via the LOCPRIOR model. This suggests weak but significant genetic structure and high levels of gene flow between subpopulations of lignum in the northern MDB. Furthermore, pairwise genetic distances as defined by *F*_ST_ values indicate that the two subpopulations not genetically differentiated and located the furthest away from each other were located at a distance of 456 km (Table 4), suggesting that gene flow occurs regularly at this distance.

Gene flow in plants at distances such as identified in this study has largely been reported for northern hemisphere forest trees such as pine and birch species as a result of wind‐mediated dispersal of pollen (Kremer et al., 2012). Pollen has been reported to have been transported by wind as far as 3,000 km (Campbell, McDonald, Flannigan, & Kringayark, 1999) from its source population, and effective pollen dispersal that results in the production of seeds and seedlings has been recorded at a distance of 600 km (Kremer et al., 2012; Varis, Pakkanen, Galofré, & Pulkkinen, 2009). Seeds can also be dispersed by wind, and there have been instances of dispersal ranging in the hundreds of kilometers (vanden Broeck et al., 2014). This indicates that gene flow via wind‐dispersed seed and pollen is possible at scales of hundreds of kilometers; however, higher levels of genetic structure than are found in this study are generally reported for Australian riverine species even at smaller distances (Hevroy et al., 2017; Prentis & Mather, 2008).

Lignum seeds are small and lightweight so movement by wind may be possible. In addition, the largely dry and flat environment in which the species occurs would also facilitate dispersal via wind. However, wind dispersal of seeds generally occurs over much smaller distances than wind‐mediated dispersal of pollen (Heuertz, Vekemans, Hausmann, Palada, & Hardy, 2003) and the lack of any obvious adaptations to wind dispersal in lignum seeds suggests that, outside of an extreme weather event, any dispersal of seeds by wind in the species would not be over significant distances (i.e., greater than a kilometer). In this study, the direction of prevailing winds was used as a measure of wind‐mediated dispersal and gene flow. However, this was not found to explain a significant proportion of the pairwise genetic differentiation between subpopulations, indicating that either wind was not a significant factor or that the prevailing winds, denoted by the angle between pairwise subpopulations, did not effectively represent wind as a factor. It is possible that the extremely low levels of genetic differentiation between distant subpopulations and associated long‐distance gene flow observed in this study are the result of underestimation of levels of genetic variation resulting from relatively low sample sizes. Despite this, the weak patterns of genetic differentiation that have been detected provide valuable insight into an ecologically important but under studied species for which genetic information is extremely limited.

AMOVA revealed highly significant differences between subpopulations within river systems, between subpopulations among river systems and among subpopulations overall (Table 3). Differences between subpopulations among river systems were smaller than differences among subpopulations within river systems. This indicates that the pattern of genetic variation of lignum subpopulations in the northern MDB does not conform to the SHM suggesting that dispersal is not limited to the stream corridor, and overland dispersal is a common occurrence. In addition, Mantel tests between genetic distance and geographic as well as river distance were both significant. However, the amount of variation that could be explained by geographic distance (0.09) was twice that of river distance (0.044), further suggesting that overland dispersal occurs more frequently than dispersal along the river corridor (Figures 3 and 4). Overland dispersal and gene flow in lignum have the potential to occur as the result of the dispersal of any of three different propagule forms, that is, seeds, pollen, and plant fragments (Chong & Walker, 2005; Roberts & Marston, 2011).

Very little is known regarding mechanisms of pollen dispersal in lignum; however, wind and insects have been suggested as possible vectors (Casanova, 2015). In addition, observations of floral visitors in the field (flies beetles and tiger moths in particular) suggest that the species may be largely insect‐pollinated. While there is record of long‐distance insect‐mediated dispersal of pollen in the order of hundreds of kilometers (Ahmed, Compton, Butlin, & Gilmartin, 2009), long‐distance dispersal of pollen by insects usually ranges from hundreds of meters to a few kilometers (Jha & Dick, 2010; Millar, Coates, & Byrne, 2014; Noreen, Niisalo, Lum, & Webb, 2016; White, Boshier, & Powell, 2002).

Other more widely traveled animals that may play an important role as vectors of overland dispersal include waterbirds that travel long distances to take advantage of the boom in productivity that occurs in these areas during times of expansive flooding. Lignum is the preferred nesting habitat for many of these waterbird species (Kingsford, Thomas, & Curtin, 2001; Maher & Braithwaite, 1992) as they take advantage of the protection of the surrounding floodwaters and the thin intertwining branches of lignum clumps that make them perfect nesting substrate. Birds and other animals can transport propagules in two ways: Endozoochory refers to seeds dispersed through ingestion by an animal; and ectozoochory refers to seeds or pollen dispersed by seeds that, for a period, are attached to the outside of an animal. The important role of birds in dispersing plant propagules both in terrestrial and in aquatic environments has long been recognized (Green & Emberg, 2014; van Leeuwen, van der Velde, van Groenendael, & Klaasen, 2012; Sekercioglu, 2006). In particular, their potential contribution to gene flow in ephemeral freshwater habitats such as those of dryland rivers and wetlands in Africa and Australia has recently been examined (Green, Jenkins, Bell, Morris, & Kingsford, 2008; Reynolds & Cumming, 2016). As a result of the long distances they travel (particularly migratory species), birds are considered important vectors of long‐distance propagule dispersal for plants with potential dispersal distances of hundreds even thousands of kilometers frequently reported (Soons, van der Vlugt, van Lith, Heil, & Klaasen, 2008; Taylor, 1954; Viana, Santamaría, Michot, & Figuerola, 2013). In an extreme case, a study of island *Acacia* species found evidence of a bird‐mediated dispersal event of around 18,000 km from Reunion Island (east of Madagascar) to the Hawaiian Islands, USA (Le Roux et al., 2014).

While lignum does not have any specific adaptations to dispersal by birds, the frequent use of the plant for refuge and nesting habitat by waterbirds, as well as the small size of its seed, indicates that ectozoochoric dispersal of seeds and pollen that become stuck in feathers and other parts of the body may be an important and frequent means of long‐distance dispersal and gene flow in the species. In addition, the small size of the seeds might result in accidental ingestion of seeds and endozoochoric dispersal. However, the ability of lignum seeds survive gut passage is unknown. Feral goats were frequently observed grazing on lignum in the field and feral pigs are also known to use lignum stands for cover, suggesting that both feral and domestic ungulates may also be vectors of dispersal. The dispersal of stem fragments could also occur in the manner described here although multilocus genotypes were not recorded so vegetative dispersal could not be confirmed.

A less obvious but probably more plausible explanation for frequent overland dispersal and gene flow indicated by patterns of genetic variation in this study is dispersal via floodwater. Hydrochory is a dispersal mechanism frequently associated with long‐distance dispersal and gene flow in riparian plant species (Fér & Hroudová, 2008; Johansson et al., 1996; Merritt, Nilsson, & Jansson, 2010; Nilsson et al., 2010; Perdereau, Kelleher, Douglas, & Hodkinson, 2014). Although none of the hydrological variables were identified as significant by the Mantel or partial Mantel tests, there are patterns in the data that suggest that hydrochory is important especially for overland dispersal. PCoA and STRUCTURE analysis showed strong grouping of the Lower Balonne sites despite them all occurring on different rivers with all subpopulations showing strong assignment to cluster 1 (Figures 2 and 5). Pairwise *F*_ST_ confirmed the grouping of these sites with none of them being significantly differentiated from each other. While some of the Warrego and Paroo subpopulations show mixed ancestry in the STRUCTURE analysis, and overall, they are not as tightly grouped on the PCoA plot they also do not exhibit significant pairwise genetic differentiation. On the Darling River, however, the Trilby subpopulation differed from both the other two subpopulations.

The increased genetic similarity between Warrego and Paroo River subpopulations and subpopulations located on the rivers of the Lower Balonne could be a result of increased hydrochoric dispersal across shared floodplains. A similar pattern was identified for an endangered shrub in Western Australia, albeit over much smaller distances. Although unlike this study, Hevroy et al. (2017) found greater differences among rather than within drainage lines, differences among floodplain populations were smaller than both those recorded within drainage lines and among drainage lines even at similar distances. They proposed that episodic flood events, although less frequent than creek line flow, provide for more effective dispersal of seeds that is independent of distance. In the current study, Darling subpopulations were more differentiated than the other river systems despite being located on the same floodplain adjacent to the main channel. The difference between subpopulations on this river in comparison with the lack of differentiation between subpopulations on the other rivers that shared floodplains could be attributed to differences in channel morphology and flow regime. In comparison with the other rivers in this study, the main channel of the Darling is very deeply cut with high banks and while Darling River gauging stations had the lowest proportion of no flow days this did not translate to increased frequency of high flow events (Table 2). In comparison, rivers in the Lower Balonne as well as the Warrego and Paroo have relatively shallow channels and although they generally have higher proportions of days with no flow they also have similar or greater frequencies of high flow events (Table 2). Given its high banks, comparatively high flow events are likely to be required for significant overbank flooding in the Darling, and this means that overbank flows required for dispersal of seeds via hydrochory over the floodplain are likely to be rare and successive events essential to the successful establishment seedlings even rarer. Further, support for the presence of overland dispersal via floodwater is evident in the similar assignment proportions and lack of significant pairwise genetic differentiation between Enngonia and Lagoon populations which are connected by a common floodplain that is likely to be inundated during significant flow events and the same similarities between Weir and Corni‐Paroo subpopulations which are essentially connected by a small ephemeral channel (Cuttaburra Creek) that directs flood waters during high flow events (Figure 1).

## 5 CONCLUSIONS

In conclusion, the results of this study indicate that gene flow is high between subpopulations for lignum in the northern MDB. Frequent long‐distance dispersal events are likely, as indicated by weak levels of spatial genetic structure. The exact mechanisms of long‐distance dispersal are not clear; however, the presence of greater genetic differentiation between subpopulations within river systems in comparison with subpopulations among river systems indicates nonconformity with the SHM and suggests that dispersal is not limited to the river corridor. Despite this, patterns of pairwise genetic differentiation and clustering according to STRUCTURE suggest a pattern of genetic variation best explained by floodplain dispersal via hydrochory. This is in agreement with experimental data finding that lignum seeds can germinate while floating and achieve optimal germination when inundation occurs for around 20 days before seeds are deposited on to wet soil (Chong & Walker, 2005; Higgisson et al., 2018). Despite the apparent importance of dispersal via floodwater, flow variables did not significantly explain genetic variation. This is probably because these variables were designed to capture in channel conditions important if dispersal was occurring along the river corridor rather than across the floodplain as was found to be more common. Spatial patterns of genetic variation in the species, however, cannot solely be explained by hydrochory, and dispersal of seeds and possibly pollen by nesting waterbirds as well as pollination by insects are also likely to be important.

This study highlights the importance of maintaining natural flow variability in these systems as overbank flows are clearly integral to the persistence of this species not just for the provision of conditions that promote germination and establishment but also to facilitate gene flow and dispersal across the floodplain and between river channels. While rainfall predictions under future climate change scenarios are uncertain predictions for the MDB generally forecast an overall drying effect with an increase in the intensity of rainfall events and the prolonging of intervening drought events (James, Reid, & Capon, 2016). This in mind the persistence of floodplain plants such as lignum in these environments may be more reliant on their ability to endure drought periods of increasing duration and respond quickly to unpredictable wet periods than on efficient dispersal capacity. While the patterns of weak genetic differentiation and population structure described here provide valuable insight into a keystone floodplain plant, relatively small sample sizes suggest the results should be treated with caution. Further studies involving more individuals are required to confirm the high levels of gene flow suggested by the current data. In addition, the use of detailed flood frequency maps may be useful in determining the exact nature of overland dispersal and gene flow via hydrochory.

## CONFLICT OF INTEREST

None declared.

## AUTHOR CONTRIBUTION

BM, MR, SC, and SW conceived the ideas; BM collected data; BM and SW performed laboratory analysis; BM and MR performed statistical analysis; BM led the writing; and MR, SC, and SW revised drafts for important intellectual content.

## Data Availability

Microsatellite sequence data have been submitted to GenBank under the accession numbers KX762272–KX762283 (www.ncbi.nim.nih.gov/genbank).
